# Sleep-Dependent Synaptic Down-Selection (II): Single-Neuron Level Benefits for Matching, Selectivity, and Specificity

**DOI:** 10.3389/fneur.2013.00148

**Published:** 2013-10-04

**Authors:** Atif Hashmi, Andrew Nere, Giulio Tononi

**Affiliations:** ^1^Department of Electrical and Computer Engineering, University of Wisconsin-Madison, Madison, WI, USA; ^2^Department of Psychiatry, University of Wisconsin-Madison, Madison, WI, USA

**Keywords:** neurons, plasticity and learning, sleep, homeostatic regulation, information

## Abstract

In a companion paper (1), we used computer simulations to show that a strategy of activity-dependent, on-line net synaptic potentiation during wake, followed by off-line synaptic depression during sleep, can provide a parsimonious account for several memory benefits of sleep at the systems level, including the consolidation of procedural and declarative memories, gist extraction, and integration of new with old memories. In this paper, we consider the theoretical benefits of this two-step process at the single-neuron level and employ the theoretical notion of *Matching* between brain and environment to measure how this process increases the ability of the neuron to capture regularities in the environment and model them internally. We show that down-selection during sleep is beneficial for increasing or restoring *Matching* after learning, after integrating new with old memories, and after forgetting irrelevant material. By contrast, alternative schemes, such as additional potentiation in wake, potentiation in sleep, or synaptic renormalization in wake, decrease *Matching*. We also argue that, by selecting appropriate loops through the brain that tie feedforward synapses with feedback ones in the same dendritic domain, different subsets of neurons can learn to specialize for different contingencies and form sequences of nested perception-action loops. By potentiating such loops when interacting with the environment in wake, and depressing them when disconnected from the environment in sleep, neurons can learn to match the long-term statistical structure of the environment while avoiding spurious modes of functioning and catastrophic interference. Finally, such a two-step process has the additional benefit of desaturating the neuron’s ability to learn and of maintaining cellular homeostasis. Thus, sleep-dependent synaptic renormalization offers a parsimonious account for both cellular and systems level effects of sleep on learning and memory.

## 1 Introduction

In a companion paper (1), we showed with simulations of simple neuronal systems that an activity-dependent mechanism of synaptic potentiation in wake and off-line depression in sleep can account for several benefits of sleep on memory. At the systems level, these benefits include the consolidation of procedural and declarative memories, gist extraction, and the integration of new with old memories.

In this paper, we consider the benefits of this two-step activity-dependent process of synaptic potentiation in wake and off-line depression in sleep at the level of the single neuron. We start from the fundamental energy constraint that neurons should reserve *firing* for rare events and use *not firing* as a default state. As argued elsewhere (2–5), this asymmetry forces neurons to communicate important events by firing more, rather than less. In turn, this leads to the requirement that, during wake, suspicious coincidences, presumably originating from the environment, should be learned by strengthening, rather than weakening, connections. As in Nere et al. (1), we assume that neural circuits learn to both capture and model the statistical structure of the environment, which can be done by strengthening clusters of feedforward and feedback connections in the same dendritic domain.

However, if left unchecked, the progressive increase in synaptic strength imposed by the requirement of plasticity in a changing world can lead to negative consequences. These include capturing and modeling spurious (noisy) coincidences picked up from the environment, leading to progressive interference. Moreover, the selectivity of neuronal responses to suspicious coincidences decreases, along with response specificity of different subsets of neurons. Also, a neuron’s ability to learn new coincidences soon becomes saturated, and there are major consequences on cellular homeostasis. For these reasons, as argued by the synaptic homeostasis hypothesis (SHY) of sleep function (2–4), neurons need periods in which they are disconnected from the environment (off-line) and can undergo an activity-dependent process of synaptic down-selection. As illustrated in the companion paper (1), one way to do so is for neurons to reduce synaptic strength in an activity-dependent manner during sleep. In this process, strongly activated clusters of feedforward and feedback synapses, presumably reflecting regularities in the environment that fit well with previously acquired knowledge, can be protected. By contrast, weakly activated clusters of synapses, presumably reflecting spurious (noisy) coincidences that fit less with old memories, whether picked up from the environment or internally generated, can be down-selected. In this way, response selectivity and specificity are restored, learning ability is desaturated, and cellular stress is reduced.

To illustrate the benefits of this two-step process of potentiation in wake and down-selection in sleep in a principled manner, we make use of the notion of *Matching* between a system and its environment (6–8), which reflects how well a neural system captures the statistical structure of its environment (deviations from independence, i.e., suspicious coincidences) and models it internally. Using simple examples, we show that on-line synaptic potentiation in the wake phase, followed by off-line activity-dependent down-selection during sleep can ensure high levels of *Matching*. We also argue that, by a proper arrangement of connections in different dendritic compartments, different subsets of neurons can learn to specialize for different suspicious coincidences and form sequences of nested perception-action loops. By systematically potentiating such loops when interacting with the environment in wake, and depressing them during sleep when disconnected from the environment, neurons can learn to match the long-term statistical structure of the environment while avoiding spurious modes of functioning and catastrophic interference.

## 2 Materials and Methods

In this paper, we perform simulations at the level of a single integrate-and-fire neuron. In the sections below, we describe the neuron model, potentiation and down-selection mechanisms, the input distributions to the modeled neurons, and the measure of *Matching*.

### 2.1 Neuron model

As in the companion paper (1), we assume a simple integrate-and-fire neuron model with binary outputs. Similarly, the synaptic inputs to the integrate-and-fire neurons are organized into *dendritic compartments*, composed of one feedforward and one feedback synapse each. This neuron model was chosen such that our simulations would capture the constraint of requiring both feedforward and feedback activations to trigger synaptic plasticity. We note that these choices for the neuron and synapse models make a number of broad simplifications. However, in the context of this work, simple models are chosen to explicitly focus our investigation on the role of a two-step learning process on the formation and modification of memories in neuron-like elements. In future work, we plan to extend this analysis to models of higher biological fidelity.

**Figure 1:**
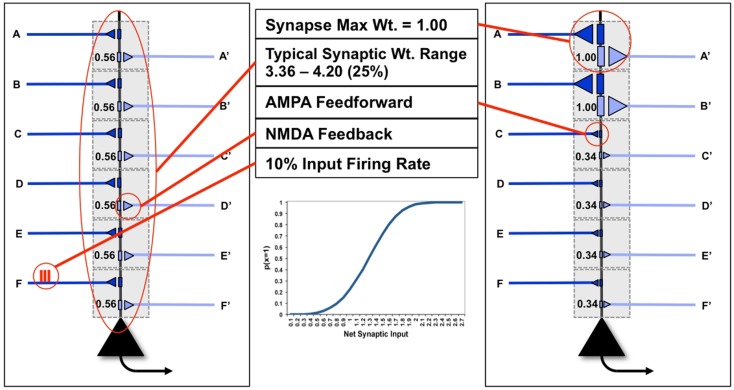
**“Uniform” and “specialized” neurons and their parameters**. The neuron on the left shows “uniform” synapses, while the neuron on the right has become “specialized” for coincident inputs on synapses A/A′ and B/B′. The inset shows the sigmoid firing probability of modeled neurons.

In Figure 1, we see two examples (“uniform” neuron on the left, “specialized” neuron on the right) of the neuron model used in this paper, with six dendritic domains, each outlined in gray. In the figure, dark blue connections indicate feedforward connections, i.e., those activated ultimately by inputs from the environment (synapses A–F). Light blue indicates feedback connections, i.e., those activated primarily by inputs generated from higher levels within the brain (synapses A′–F′). In this paper, the probability of a neuron *x* firing for a particular input *y*, *p*(*x* = 1|*y*), is a sigmoid function of the synaptic weights *W* and of the *ON* (1) or *OFF* (0) state of its inputs *y_j_*:

(1) $$\begin{array}{ll} p\left(x=1|y\right) & =1-e^{-z^{4}}∕4 \end{array}$$

(2) $$z=\sum_{j=1}^{J}W_{j}y_{j}$$

*J* is the number of inputs to a neuron (here, dendritic domains). The inset illustrates the behavior of this sigmoid. As the neuron has only two possible outputs (1 or 0) the probability that a neuron does not fire given the input is simply *p*(*x* = 0|*y*) = 1 − *p*(*x* = 1|*y*).

### 2.2 Constraints on activity and plasticity

To take into account the energy constraint that firing is more expensive than not firing (9), in the following analysis we assume that a neuron can afford to fire approximately 10% of the time [*p*(*x* = 1| = 0.1)], meaning that firing is sparse (Figure 1). Though the output firing rate of the neuron changes during the experiments described below, the firing rate on any input synapse is constrained to exactly 10%. Furthermore, neurons necessarily have cell-biological constraints over both the maximum strength of a single synapse, as well as the total amount of synaptic strength distributed over its synapses (Figure 1). In the following analysis, to keep the examples and computations as simple as possible, we consider only 6feedforward (primarily AMPA) and 6feedback connections (primarily NMDA, see Nere et al. (1)), and impose limits on individual and total synaptic strengths: each individual synaptic weight is limited to the range [0, 1]; total synaptic weight at baseline (after synaptic down-selection in sleep) is limited to the range [3.03, 3.71] (±10% of the optimal total synaptic weight for the initial example of Figure 5A, as will be described below); total synaptic weight after training is allowed to increase by 25% over baseline, resulting in a range of [3.79, 4.64], as suggested by molecular and electrophysiological data (10). In Figure 1, the schematic neuron on the left has uniform connection strength at all synapses, whereas the neuron on the right has two strong synapses (A and B) and four weak ones, for the same total synaptic weight.

### 2.3 Plasticity mechanisms in wake

**Figure 2:**
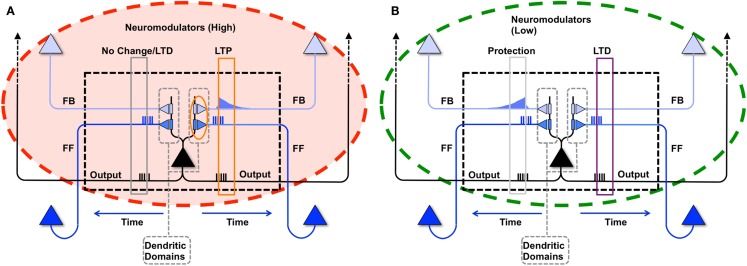
**Synaptic potentiation in wake and down-selection in sleep**. **(A)** During wake, plasticity is dominated by potentiation. Synapses are potentiated when a neuron receives persistent feedforward activation and longer timescale feedback activations on the same dendritic domain, the neuron itself exhibits strong activation, and global neuromodulators are present. The orange box indicates the dendritic domain which meets these requirements for LTP. Conversely, the gray box indicates a dendritic domain which is missing feedback activity, so either no change happens or LTD is induced. **(B)** In sleep, global neuromodulators are largely absent. The synapses in a dendritic domain are protected when the neuron is strongly activated by matching feedforward and feedback activations (gray box, left dendritic domain). Conversely, LTD occurs when a neuron fires but its feedforward and feedback are mismatched within a dendritic domain (purple box, right dendritic domain).

As described in the companion paper (1), during wake a modeled neuron potentiates its synapses within a dendritic domain dependent on the following conditions: strong feedforward firing, relayed by driving primarily AMPA connections; strong feedback firing, relayed by modulatory, primarily NMDA connections (11); strong firing of the neuron itself, suggesting that it has received strong, coincident firing on its inputs; and high levels of global neuromodulators (12, 13), gating learning in wake to salient interactions with the environment (Figure 2A). In the present simulations, although the sigmoid activation function introduces some indeterminism, a neuron is more likely to fire the more of its input synapses convey spikes simultaneously, i.e., when they detect suspicious coincidences. Under the constraint of sparse firing mandated by energy constraints, such suspicious coincidences reflect the occurrence of events that happen more frequently than expected by chance, which here are assumed to be due to the causal structure of the environment (14). Coincident firing is also required between feedforward and feedback inputs. Such coincidences, if they occur when the neuron fires, suggest the closure of a loop between input and output in which the neuron may have played a causal role. It also indicates that the feedforward suspicious coincidences the neuron has captured, presumably originating in the environment, can be matched internally by feedback coincidences generated higher-up in the brain (7). The “specialized” neuron from the right panel of Figure 1 is shown again in Figure 4B to demonstrate this causal loop. The neuron receives feedforward activations from a lower area neuron, shown in dark blue, and in turn is activated. This activation propagates to higher area neurons, shown in light blue. The dashed connections to and from the higher area neurons indicate that several neural levels may interact before the top-down activations return to the “specialized” neuron, closing the loop. On the other hand, the “uniform” neuron of Figure 4A, due to its weak connections on A and B, will not reliably activate for this suspicious coincidence, and the loop may not close.

Here, to simplify the analysis and focus on a single neuron, unlike in the companion paper (1), complex feedback loops are not modeled explicitly. Instead, we assume that the output of the neuron feeds back upon itself through feedback connections. Formally, if a neuron spikes because it received strong activations on its feedforward and feedback synapses within the same dendritic domain, then the weight corresponding to a synapse *i* within the active dendritic domain is potentiated using the following rule:

(3) $$W_{i}=\left\{ \begin{array}{l} W_{i}+\alpha ,\;ify_{i}^{FF}=1\;and\;y_{\text{Domain}}>0\\ W_{i},\text{ otherwise} \end{array}\right.$$

(4) $$y_{\text{Domain}}=\sum_{m=1}^{M}y_{m}^{FF}\sum_{n=1}^{N}y_{n}^{FB}$$

That is, if a neuron spikes, then for each of its dendritic domains that receive both feedforward and feedback activations, the active synapses are incremented by α. M and N are the number of feedforward and feedback synapses within the domain, respectively. For the present simulations, the value of α is set to 0.01. For simplicity, global neuromodulators are assumed to be high throughout learning in wake, resulting in net potentiation, in line with experimental results (12, 13).

### 2.4 Down-selection mechanisms during sleep

As in the companion paper (1), synaptic potentiation in wake is balanced by synaptic down-selection during sleep, triggered by low level of global neuromodulators (12, 13). More specifically, when a neuron fires in sleep, it depresses the synapses in the dendritic domains where feedforward and feedback activations are mismatched, and it protects the dendritic domains where feedforward and feedback activations are matched. As illustrated schematically in Figure 2B, when during sleep a neuron detects suspicious coincidences, rather than potentiating the corresponding synapses, it “protects” them from depression. By contrast, synapses that are activated in isolation are not protected and thus depress progressively in the course of sleep.

As with the potentiation mechanisms for wake described above, down-selection in sleep is assumed to be confined to dendritic domains as well. Formally, when neuron spikes during sleep, but receives either only feedforward spikes or only feedback spikes on synapses within a dendritic domain, it depresses a synapse *i* in a non-active dendritic domain according to the following rule:

(5) $$W_{i}=\left\{\begin{array}{cc} W_{i}-\beta ,\; & ify_{i}^{FF}=1\;and\;y_{\text{Domain}}=0\\ W_{i},\; & \text{otherwise}\\ \; \end{array}\right.$$

That is, if a neuron spikes and has either received feedforward without feedback or feedback without feedforward activations on synapses within a dendritic domain, the active synapses in that domain are depressed by β. For our simulations, the value of β is set to be 0.001. As with the potentiation rate β, the depression rate β was selected empirically during computer simulations. We note that for the given constraints of our experiments (i.e., input firing rates, number of connections, simulation times, etc.) a fairly broad range of values for α (0.01–0.1) and β (0.001–0.1) behaved similarly.

### 2.5 Input distributions in wake and sleep

To evaluate the extent by which activity-dependent synaptic potentiation in wake and depression in sleep can account for several benefits of sleep on memory, the synapses of the two neurons shown in Figure 1 are activated with various input distributions. A representative example of such a distribution is described here. To restrict modeling to a single neuron, we assume that the feedforward input on synapse A, originating from the environment, coincide with the feedback input on synapse A′, originating in higher areas, within the same compartment (as demonstrated by the closed loop of the “specialized” neuron in Figure 1). Therefore, the number of possible input patterns over the 6compartments effectively reduces to 2^6^ = 64.

**Figure 3:**
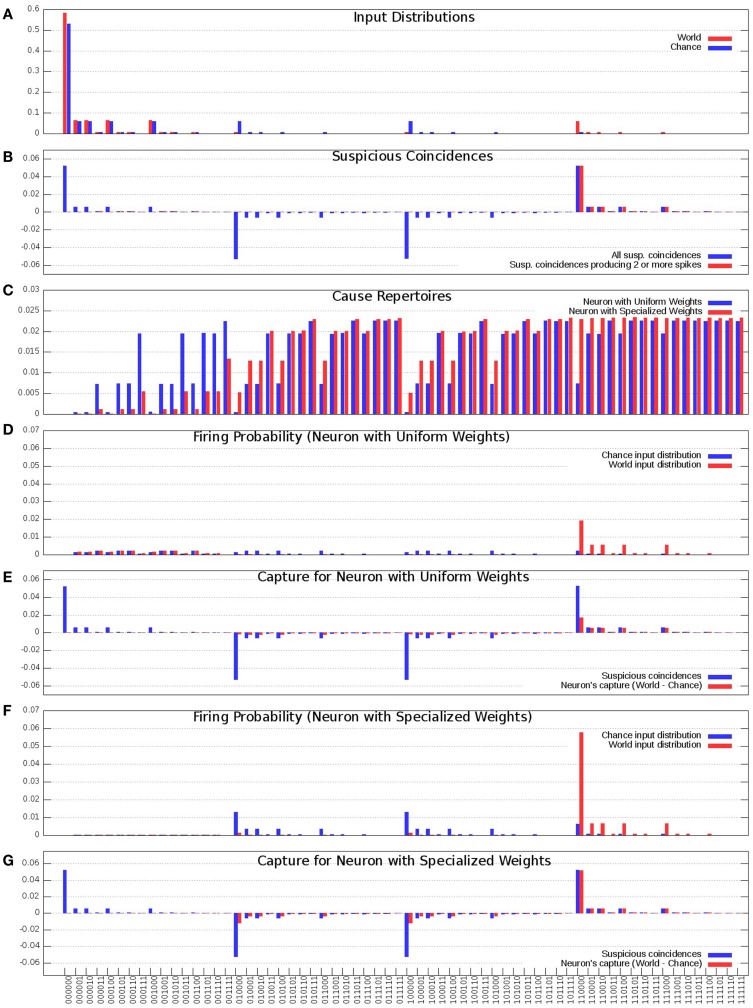
**Distributions of the “uniform” and “specialized” neurons**. **(A)** Probability of occurrence of each of the 64 input states. *Chance* (Blue) corresponds to the distribution where each input is equally likely and is active 10% of the time, *World* (Red) corresponds to the distribution where inputs A and B co-occur 9% of the time and all the inputs get active 10% of the time. **(B)** Suspicious coincidences present in the input distributions. All possible suspicious coincidences are shown in blue while positive only suspicious coincidences corresponding to two or more active inputs are shown in red. **(C)** Cause repertoires corresponding to the “uniform” and “specialized” neurons. **(D)** “Uniform” neuron’s firing probabilities in response to the *Chance* and *World* input distributions. **(E)** “Uniform” neuron’s capture (Red) compared against the suspicious coincidences (Blue). **(F)** “Specialized” neuron’s firing probabilities in response to the *Chance* and *World* input distributions. **(G)** “Specialized” neuron’s capture (Red) compared against the suspicious coincidences (Blue).

In Figure 3A, the blue bars show the input distribution corresponding to independence among the 6 inputs, where every input fires 10% of the time, reflecting the energy constraint of sparse firing. Under independence (*Chance* condition), coincidences of firing occur purely by chance (spurious coincidences), with progressively lower frequency for inputs that contain more spikes (i.e., the number of 1s in the input). Thus, more than 50% of input patterns are 000000; coincidences of two input spikes (e.g., 110000) occur 1/10*1/10 = 1% of the time, while the input pattern 111111 occurs 0.0001% of the time. In Figure 3A, the red bars show the input distribution for the *World*, which has a non-random statistical structure. In this *World*(AB), inputs A and B are 90% correlated, and hence fire together 9% of the time (suspicious coincidences). For the present purposes, the same input distribution is assumed to be generated internally during sleep (*Sleep*).

In Figure 3B, the blue bars show the difference in received spikes when the neuron is exposed to *World*(AB) as compared to when it is exposed to *Chance*. For example, input pattern 110000 (A and B both *ON*) occurs more often than would be expected by chance, whereas other input patterns, such as 100000 (A) occur less frequently than expected by chance (since most inputs on A are correlated with the input on B).

As argued in the companion paper (1), neurons should pay particular attention to suspicious coincidences of firing, which here correspond to two or more synchronous input spikes. These suspicious coincidences of firing are illustrated by the red bars in Figure 3B.

In Figure 3C, the blue bars show the *cause repertoire* for the “uniform” neuron in Figure 1 (left panel) when it is firing, i.e., the probability with which each possible input state could have made it fire (the probability of not firing is simply the complement to 1). Note that, consistent with the fact that the “uniform” neuron of Figure 1 has synapses which are all the same weight, the cause repertoire indicates that, irrespective of which particular input spikes, input patterns with just one input spiking are not likely causes of the target neuron firing, and that the probability of causing the neuron to fire increases with the number of synapses carrying spikes, saturating at about four coincident spikes. The red bars show the cause repertoire for the “specialized” neuron in Figure 1 (right panel). Note that input states with A and B firing are more likely causes of the specialized neuron firing, in line with the stronger connections on lines A and B.

In Figure 3D, the blue bars show the observed probability of firing for the “uniform” neuron of Figure 1 (left panel) in the *Chance* condition. For each input pattern, this is the joint (product) probability of that particular input and that of the neuron firing (*x* = 1) for that input. The red bars show the same for *World*(AB) condition. Clearly, the neuron is more likely to fire for input pairs that occur more frequently than expected by *Chance*(AB), but that is merely a reflection of their increased frequency of occurrence, not of the neuron being selective for those pairs. In fact, as shown in Figure 3E, the neuron’s increased firing for suspicious coincidences (in red) is not proportional to their occurrence (in blue).

Compare this with the “specialized” neuron in Figure 1 (right panel), whose connections have been selected to detect suspicious coincidences in *World*(AB) by increasing the weight of connections A/A′ and B/B′. As shown by Figure 3C (red bars), the specialized neuron is more likely than the “uniform” neuron to fire for coincident inputs on A and B. As a consequence, its observed probability of firing for input patterns AB in the *World* condition is much higher than for the neuron with uniform synaptic weights (Figure 3F). In fact, as shown in Figure 3G, the neuron’s increased firing for suspicious coincidences (in red) captures almost perfectly their frequency of occurrence (in blue).

### 2.6 Matching

Measures of *Matching* aim at quantifying how well the informational/causal structure of a system “resonates” with that of its environment (6– 8). The informational/causal structure of a system of elements is obtained by considering the set of concepts generated by subsets of elements within the system (7). A concept is the distribution of irreducible causes and effects (cause and effect repertoires) specified by each subset of elements in a state (7). When considering just one neuron, e.g., the “specialized” neuron from Figure 1 (right panel), there is only the subset corresponding to that neuron, whose cause repertoire is shown in Figure 3C (red bars, when the neuron is firing; their complement to 1, when it is silent). Each concept is associated with a value of integrated information (ϕ), which indicates to what extent the concept cannot be reduced to independent sub-concepts by partitions of the subset and their causes/effects, as measured by distance D, weighted by ϕ (e.g., Wasserstein’s metric (7)). By this definition, ϕ is higher when the neuron is firing, reflecting the greater selectivity of the causes of firing than of silence (5). The set of concepts specified by all subsets of elements of a system specify a *constellation* C of points in concept space, where each axis is a different state of the system. A constellation also has an associated value of integrated information (Φ), which indicates to what extent it cannot be reduced to independent sub-constellations (by partitions of the system, measured again by distance D, weighted by Φ (7)). Conveniently, for the single neuron considered here the constellation of concepts reduces to a single point of weight ϕ, and since a point is irreducible, in this case Φ = ϕ.

When a system interacts with its environment (*World* condition), over time it will specify a *set of constellations* C, each with an associated value of integrated information Φ. In turn, each constellation in the set can be thought of as specifying a point of weight Φ in constellation space (where each axis is a different concept specified within the system). Finally, *Matching* can be defined as the weighted distance D between the set of constellations (C) specified when the system is exposed to *World* and to *Noise*, i.e., to a structureless environment that by definition cannot be matched (here, the *Chance* condition):

(6) Matching(M)=D[C(World),C(Noise)]

where D is a distance measure weighted by Φ. Again, for the single neuron in Figure 1 (right), the set of constellations reduces conveniently to just two points, one for firing and one for silence. In general, *Matching* will be high if a system’s response to different inputs sampled from *World* is both highly differentiated (many different constellations) and integrated (each of high Φ), whereas its response to inputs sampled from Noise is not. Thus, one would expect that a complex system such as the brain, when exposed to a *World* which it is attuned to (e.g., a movie), will enter different states for different inputs, each activating many different concepts. In this way, it will specify a set C *(World)* comprised of many different constellations (indicating high differentiation), each of high Φ (indicating high integration). By contrast, when exposed to noise (e.g., a TV screen out of tune), it will enter a small number of states (in the limit, just one state), activating very few concepts. It will thus specify a set C (*Noise*) comprised of few similar constellations of low Φ. Under the assumption that a system is integrated and that similar system states generate similar constellations of concepts, *Matching* can be approximated more conveniently as the distance D, not between sets of constellations, but between the distributions of system states S, when the system is exposed to *World* and to *Noise* (7):

(7) Matching(M)=D[S(World),S(Noise)]

Estimating this distance for realistic systems requires many simplifying assumptions and computational approximations (Boly et al., in preparation; Albantakis et al., in preparation). However, due to the choice of a single integrate-and-fire neuron for the present simulations, measuring *Matching* becomes straightforward: since there is only one neuron with two states, *Matching* becomes simply a function of the difference between the distribution of firing and silence in the *World* and *Chance* conditions. The more these two distributions differ, i.e., the more the neuron fires for *World* compared to *Chance*, the higher the value of *Matching*. In what follows, this measure of *Matching* will be used to evaluate quantitatively how well the neuron’s connection weights “resonate” with its environment before learning, after learning in wake, and after down-selection in sleep, as well as to compare down-selection to other plasticity strategies.

**Figure 4:**
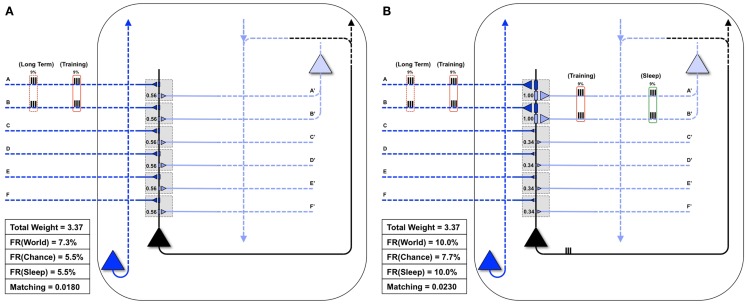
**Comparing the “uniform” and “specialized” neurons**. Both the “uniform” (A) and the “specialized” neurons (shown in black) are embedded in closed loops. Feedforward connections, ultimately generated by the outside world, are indicated by dark blue, while feedback connections, ultimately generated by higher brain areas, are indicated by light blue. The *World* has a *Long-Term* statistical structure (dashed red boxes) and a *Training* input for a particular day (solid red boxes). **(A)** The “uniform” neuron is not adapted for coincident inputs AB. As a result, it may not fire for the inputs AB, and thus will not see corresponding feedback on connections A′ and B′. *Matching* for the “uniform” neuron is low. **(B)** Conversely, the “specialized” neuron is adapted to the coincident inputs AB, fires reliably when they are active, and receives corresponding feedback on connections A′ and B′ (as shown by the red *Training* box on its light blue connections). Furthermore, in *Sleep*, coincident activations corresponding to these strong connections will be much higher than by chance (9% of the time, as indicated by the green box on the right). *Matching* for the “specialized” neuron is high.

Figure 4 shows the statistical structure of a particular environment (*World*(AB)), firing rates in the *World*, *Chance*, and *Sleep* condition, and values of *Matching* for both the “uniform” neuron (all connections of equal strength) and the “specialized” neuron (stronger connections on synapses A/A′ and B/B′). In *World*(AB), suspicious coincidences (synchronous firing above what would be expected by chance) occur on input lines A and B, as indicated in Figure 4. The “uniform” neuron in Figure 4A, having the same weight (0.56) on all forward connections, has no way of sampling preferentially the suspicious coincidences in *World*(AB) (see Figure 3). As a consequence, the neuron fires at a rate of 7.3%, which is not much higher than the rate when input patterns occur at chance (5.5%). Since we assume that feedback connections mirror feedforward ones and are thus uniform in weight, the neuron also has no way of modeling the increased frequency of occurrence of AB firing. In this case, calculating *Matching* yields 0.018. Note that the value of *Matching* for the “uniform” neuron is low but non-zero because the sigmoid function is tuned in such a way (Figure 1, inset) that the probability of firing rises sharply (inflection point) around a total synaptic input of 1.2, and any two active synapses for the “uniform” neuron sum to 1.12 (total weight of two synapses = 0.56 + 0.56 = 1.12). Two synapses being co-active happen more frequently in the *World*(AB) than in the *Chance* condition, so firing is higher with *World*(AB). However, since the “uniform” neuron has not allocated synaptic strength preferentially to connections A and B, the probability of firing for that suspicious coincidences is just around 0.5 (and not any higher).

By contrast, the “specialized” neuron in Figure 4B has allocated higher synaptic weight (1.0) to connections A and B, for a total synaptic weight of 2.0. Based on the sigmoid function governing firing, when these two synapses are active, the neuron will fire with probability near 1. Therefore, whenever inputs A and B co-occur, the “specialized” neuron is virtually certain to fire, leading to a high firing rate with *World*(AB). Inevitably, due to increased total synaptic weight on these two synapses, the firing rate under *Chance* will also be higher. However, crucially, the resulting increase in firing rate is much more likely to occur with *World*(AB) than with *Chance*, and thus *Matching* is higher for the “specialized” neuron (0.023). These simple examples illustrate that *Matching* can be used to evaluate to what extent a system’s architecture (here, the weight of the synapses on a single neuron) can sample the statistical structure of its environment as well as model it. Below, *Matching* will be employed to measure how learning by potentiation in wake, followed by down-selection in sleep, can benefit memory consolidation, and how alternative schemes may not work as well.

We note here that, in the following experiments, an “ideal” value for *Matching* is not defined. Primarily, this is because the value of *Matching*, as defined in this paper, is determined by the difference between a neuron’s response to a set of coincidences (*World*) and noise (*Chance*) within the context of the chosen simulation constraints (i.e., total synaptic weight, maximum synapse value, maximum input firing rate, etc.). Therefore, the maximum possible value for *Matching* will be different for a *World*(AB) and a *World*(CDE). Rather, in the following experiments, we examine the changes in *Matching* during potentiation in wake and down-selection in sleep, but do not compare the values of *Matching* across different experiments.

## 3 Results

Given the set of constraints outlined above, we now present several examples to analyze how *Matching* changes as a neuron potentiates synapses during wake and depresses them in sleep.

### 3.1 Sleep-dependent synaptic down-selection improves matching

**Figure 5:**
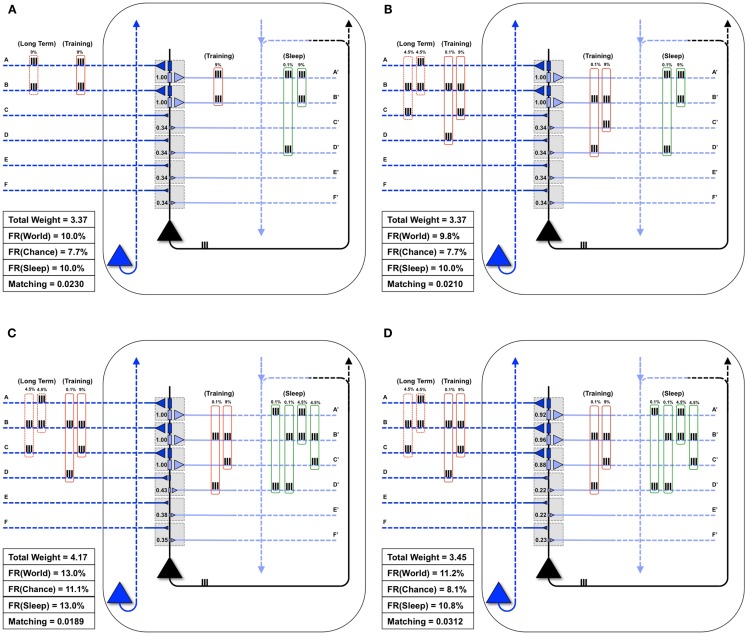
**Sleep-dependent synaptic down-selection improves *Matching***. **(A)** Initially, the *World* shows statistical correlation on inputs A and B, and the neuron has appropriately strengthened synapses A/A′ and B/B′. Thus, *Matching* is high. **(B)** The *Long-Term* statistics of the *World* change such that inputs on A and B, as well as B and C are correlated (as indicated by the dashed red boxes on the left). On this particular *Training* day, the neuron is exposed to correlated inputs B and C, as well as to a small percent of spurious coincidences on inputs B and D. Before synaptic potentiation occurs, *Matching* is reduced since the neuron does not yet capture the statistics of the *World*. **(C)** During wake, the neuron potentiates synapses C/C′, while synapses D/D′, E/E′, and F/F′ are only slightly strengthened. The neuron now responds appropriately to the *World*, but the extra synaptic weight also means its response to *Chance* goes up even more. As a result, *Matching* is further reduced. **(D)** During sleep, the neuron often reactivates inputs on A and B (4.5%), B and C (4.5%), but also spurious inputs on A and D (0.1%) and B and D (0.1%), as indicated by the green boxes on the right. Since down-selection is activity-dependent, synapses A/A′, B/B′, and C/C′ are best protected, while other synapses are depressed. As a result, the neuron still responds well to the *World*, but less so to *Chance*, thus improving *Matching* to the statistical distribution of the *World*.

Figure 5A shows the same “specialized” neuron as in Figure 1 (right panel) and Figure 4B, which is well-matched to *World*(AB). For a first experiment, we assume that the statistical structure of *World* has changed to AB, BC, and the neuron must learn the new coincidence (BC). Reflecting the appearance of this new coincidence, during a particular waking day, inputs B and C are co-activated 9% of the time (Figure 5B). There is some noise, illustrated here by spurious coincidences of firing, 0.1% of the time, between inputs B and D. These values were chosen so that the total amount of synchronous input activity was always near 9%. All other inputs were taken to be independent (as in *Chance*) and were active 10% of the time. The dashed red boxes on the left indicate the long-term statistical structure of *World* inputs (AB, BC), the solid red boxes that of the short-term inputs to the neuron during one episode of wake (BC, training), which in this case is taken to be the same. On the right hand side, the green box indicates the input to the neuron during sleep (*Sleep*). Since the synaptic weights have not yet changed to properly capture and model this new *World*(AB, BC), *Matching* is only 0.021 (Figure 5B).

Next, as in Nere et al. (1), we simulated wake training by applying inputs with the appropriate short-term statistical structure while plasticity was enabled in the wake mode, within the overall constraints of maximum individual and total synaptic strength indicated in the Section “Materials and Methods.” As shown in Figure 5C, training led to a potentiation of both feedforward and feedback synapses that sampled suspicious coincidences in the input, especially synapses C/C′, but also, due to the occurrence of spurious coincidences, synapses D/D′. As a consequence of this overall strengthening of synapses, the neuron’s firing rate increases both with *World* (FR(*World*) = 13%) and even more with *Chance* (FR(*Chance*) = 11.1%), so that *Matching* shows a slight decrease, which reflects a decrease in the neuron’s selectivity for suspicious coincidences.

As before, we assume that feedback connections changed the same way as feedforward ones, and that circuits upstream in the brain would be able to provide *Sleep* inputs to the neuron that resemble the long-term statistics of *World*(AB, BC) (green boxes in the Figure). The *Sleep* inputs to the neuron also include two infrequent “spurious” coincidences, AD and BD, which are co-activated 0.1% of the time each. We simulated spontaneous activity during sleep accordingly, while enabling synaptic down-selection. As shown in Figure 5D, the result was that, while synapses A/A′, B/B′, and C/C′ were slightly depressed, the other synapses were depressed much more markedly, including synapses D/D′. After sleep-dependent down-selection, the neuron’s firing rates with *World* decreased (to 11.2%), but it decreased more markedly with *Chance* (to 8.1%), leading to an increase in *Matching*.

### 3.2 Extra wake degrades matching

**Figure 6:**
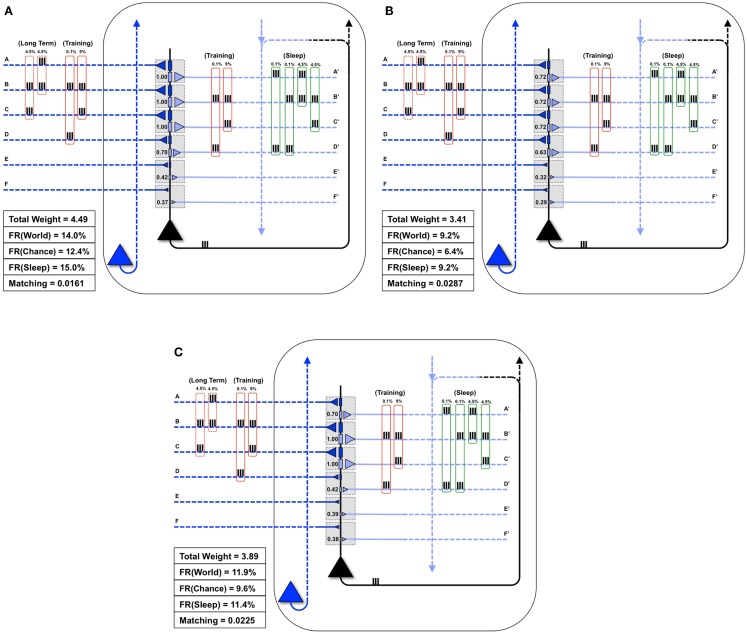
**Extra wake, potentiation in sleep, and down-selection in wake degrade *Matching***. **(A)** The same neuron shown in Figure 5C is allowed to undergo an extended period of training. The constraint on the total synaptic weight is relaxed. As a result, the neuron continues to potentiate synapses D/D′, E/E′, and F/F′. While the firing rate in response to the *World* increases slightly, the firing rate in response to *Chance* increases more significantly, thus degrading *Matching* **(B)** The same neuron shown in Figure 5C is allowed to undergo synaptic potentiation during sleep, using the correlated inputs generated internally (shown in the green boxes). Since synapses A/A′, B/B′, and C/C′ are saturated before this sleep period, the synapses D/D′ corresponding to the spurious activations (A and D 0.1%, B and D 0.1%) become further potentiated. Afterward, all synapses are linearly depressed so the total synaptic weight after sleep is comparable to the neuron shown in Figure 5D. As a result, *Matching* is degraded. **(C)** The neuron from Figure 5B is allowed to undergo both synaptic potentiation as well as activity-dependent down-selection in wake. Since the *Training* of this particular day does not include correlations on inputs A and B (but they are still present in the *Long-Term* statistics of the *World*), synapses A/A′ become depressed. Thus, *Matching* is degraded.

Figure 6A illustrates what happens if, instead of undergoing activity-dependent down-selection during sleep, the neuron from Figure 5C undergoes an equivalent period of wake during which it continues to be exposed to the new feature (BC). For this experiment, the constraint of the total synaptic weight was relaxed, allowing total weight to increase up to a value of 4.49. Predictably, synapses D/D′ continued to be strengthened due to the spurious coincidence BD, and the synaptic strength of E/E′ and F/F′ increased slightly due to occasional co-firing under independence. Even more than after a regular period of wake (Figure 5C), extended wake led to a further increase in firing rate with *World* (FR(*World*) = 14%). However, the change in firing rate was even more pronounced with the *Chance* condition (FR(*Chance*) = 12.4%). Consequently, *Matching* decreases further to 0.0161, reflecting a reduction in the neuron’s selectivity for suspicious coincidences, the opposite of what is observed if the neuron is allowed to undergo down-selection in sleep.

### 3.3 Synaptic potentiation in sleep degrades matching

Figure 6B shows instead what happens if, after training in wake, the neuron is allowed to undergo synaptic potentiation while being exposed to spontaneous activity during sleep. That is, instead of the down-selection rule (Figure 2B), plasticity during sleep is made to mirror the potentiation rule as in wake (Figure 2A). As in the previous experiment, the constraint of the total synaptic weight was relaxed, allowing total weight to increase up to a value of 4.49. In line with experimental data on reactivation (15–17), it is assumed that spontaneous activity during sleep, while partly “replaying” patterns of activity observed during wake training, is “noisy.” This is because spontaneous activity is not constrained by environmental inputs and includes many additional constraints reflecting prior knowledge incorporated in the network. Thus, in *Sleep*, the spontaneously generated correlation structure is assumed to be 4.5% AB, 4.5% BC, 0.1% AD, and 0.1% BD (as shown in the green boxes of Figure 6B). As a consequence, while synapses A/A′, B/B′, and C/C′ are further potentiated, D/D′ is also markedly strengthened, and E/E′, and F/F′ also increase slightly due to chance coincidences. Finally, in line with the evidence that total synaptic strength is reduced after sleep (2–4), the synapses were downscaled such that the total synaptic weight was 3.41 (to be comparable to the total synaptic weight for the neuron which underwent activity-dependent down-selection in Figure 5D). As shown in Figure 6B, *Matching* is reduced as compared to when sleep led to down-selection.

### 3.4 Synaptic depression in wake degrades matching

Finally, Figure 6C shows what happens if down-selection is allowed to happen during a wake episode, when the neuron is exposed to a limited sample of the environment (BC). As one would expect, training on coincident inputs (BC) with both synaptic potentiation and down-selection enabled results in a significant depression of synapses A/A′. This is because the wake-training input for this particular “day” does not include the input AB, even though the long-term statistics of this *World* are AB, BC (as shown in the dashed red boxes). As a consequence, after training with down-selection in wake, *Matching* is only 0.0225. This is markedly less than the experiment in Figure 5D, with potentiation in wake and down-selection in sleep, where *Matching* was 0.0312.

### 3.5 Synaptic down-selection in sleep favors new memories that fit with old memories

**Figure 7:**
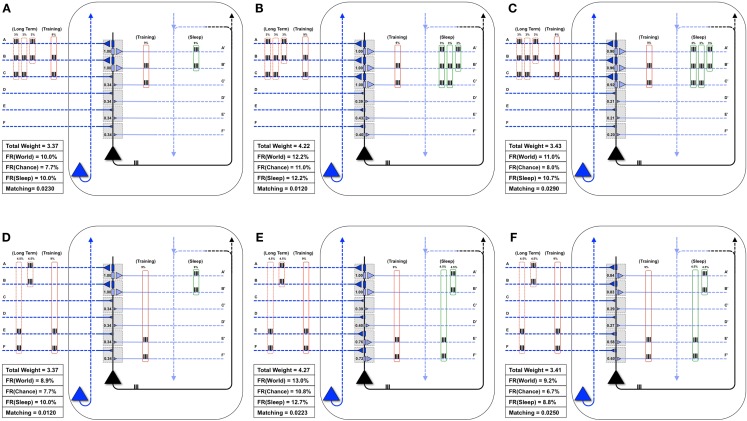
**Overlapping memories are better integrated by synaptic down-selection**. **(A)** The neuron has initially potentiated synapses A/A′ and B/B′, while the *Long-Term* statistics of the *World* show overlapping correlations AB (3%), BC (3%), and ABC (3%) (shown in the dashed red boxes on the left). During *Training*, the neuron is exposed to correlations BC (9%) (shown in the solid red boxes). **(B)** After training in wake, the neuron potentiates synapses C/C′, though *Matching* is slightly degraded by the increase in synaptic weight (which results in a greater response to *Chance*). **(C)** During activity-dependent synaptic down-selection in sleep (with the inputs shown in the green boxes on the right), synapses A/A′, B/B′, and C/C′ are often active together, and thus well protected. As a result, *Matching* improves. **(D)** Another neuron also starts with strong synapses A/A′ and B/B′. The *Long-Term* statistics of the *World* show disjoint correlations AB (4.5%) and EF (4.5%) (shown in the dashed red boxes on the left). During *Training*, the neuron is exposed to correlations EF (9%) (shown in the solid red boxes). **(E)** After training in wake, synapses E/E′ and F/F′ have been potentiated. **(F)** During activity-dependent synaptic down-selection in sleep (with the inputs shown in the green boxes on the right), inputs on A and B are activated independently of inputs on E and F. As a result, both pairs of synapses become slightly more depressed than for the neuron showing overlapping reactivation of inputs from **(C)**. As a result, *Matching* is worse than for the neuron with overlapping inputs/memories (Figure 6C).

Next, as in Nere et al. (1), we consider the integration of new with old memories. The neuron in Figure 7A is embedded in a *World* whose long-term structure is such that inputs A and B are active together 3% of the time, B and C 3% of the time, and A, B, and C are active together 3% of the time. Initially, the neuron has strong weights only on synapses A and B, and *Matching* is 0.023. On a particular wake episode, the neuron is exposed to new coincident inputs B and C, which are active together 9% of the time. In this case the “new” memory (B, C) has an overlap with the “old” memory (A, B). As in the similar case of Section 3.1.1, training in wake leads to the potentiation of synapses C/C′ (Figure 7B) and sleep to down-selection, bringing about an increase of *Matching* (Figure 7D).

Figure 7D shows the same neuron as in Figure 7A, which is attuned to coincident inputs AB. However, in this case the long-term statistics of the world contains non-overlapping coincident inputs: AB 4.5% of the time, and EF 4.5% of the time. As in the previous cases, on this particular day the neuron only sees one new set of coincidences, EF, 9% of the time. As shown in Figure 7E, this results in a potentiation of synapses E/E′ and F/F′, and *Matching* improves. However, unlike in the previous case, during activity-dependent down-selection in sleep the new memory EF and the old memory AB do not overlap. Hence, their reactivation is modeled as occurring independently, without synergy and therefore with less protection from depression. As a consequence, *Matching* improves less (Figure 7F) than it does for the “integrated” memory in Figure 7C.

### 3.6 Synaptic down-selection increases matching by forgetting correlations no longer in the environment

**Figure 8:**
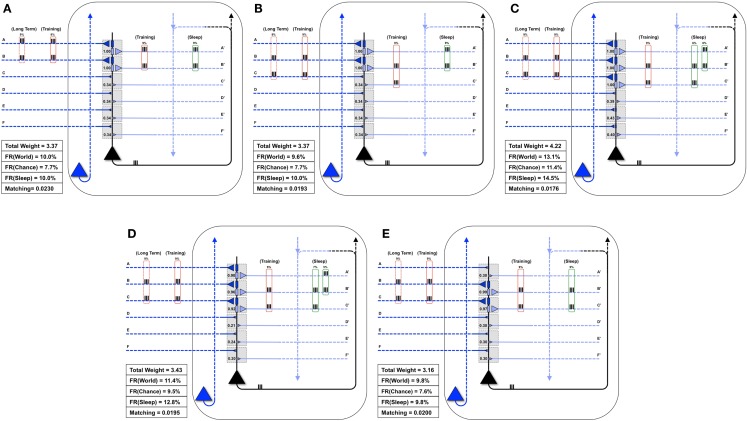
**Synaptic down-selection in sleep allows “forgetting” to improve *Matching***. **(A)** Initially, the neuron shows good *Matching* for a *World* that exhibits statistical correlations on inputs A and B. **(B)** The *Long-Term* statistics of the *World* change such that A and B are no longer correlated, but inputs on B and C are correlated (dashed red boxes on left). Since the neuron no longer fits with its *World*, *Matching* goes down. **(C)** During wake, the neuron potentiates synapses C/C′. However, as the total synaptic weight has increased, the neuron also responds more to *Chance*, and thus, *Matching* does not yet improve. **(D)** After activity-dependent down-selection in sleep, synapses A/A′, B/B′, and C/C′ are best protected, and *Matching* slightly improves. However, after a single wake/sleep period, the connection of synapses A/A′ has not yet been “forgotten.” **(E)** During subsequent wake/sleep cycles, synapses B/B′ and C/C′ are potentiated again during wake, while synapses A/A′ are not. Thus, synapses A/A′ continue to exhibit progressive depression during down-selection in sleep. After multiple wake/sleep cycles, synapses A/A′ are depressed to the same level as synapses D/D′, E/E′, and F/F′. Thus, *Matching* improves further as the strong connections for A/A′ are “forgotten.”

Finally, Figure 8A shows a neuron well-matched for *World*(AB). In this case, we assume that the long-term structure of the world changes in such a way that inputs A and B are no longer coincident, but inputs B and C are active together 9% of the time (Figure 8B). As expected, after the world has changed, but before the neuron has had a chance to adapt, *Matching* decreases from 0.0230 to 0.0193 (Figure 8B). During training in wake, the neuron potentiates synapses C/C′ (Figure 8C). However, as synapses A/A′ are still strong, they increase the neuron’s response to noise, and *Matching* is low (0.0176). After activity-dependent synaptic down-selection in sleep, synapses A/A′, B/B′, and C/C′ become slightly depressed (Figure 8D), and *Matching* improves slightly (0.0195). After multiple wake-training/sleep down-selection iterations, synapses B/B′ and C/C′ continue to be potentiated in wake to balance their down-selection in sleep, while A/A′ is only down-selected. As a result the neuron progressively “forgets” the memory of AB (Figure 8E), and *Matching* improves further (0.020).

## 4 Discussion

The above examples show how the benefits of a two-step plasticity strategy, involving net synaptic potentiation of clusters of feedforward and feedback synapses in wake, and net depression during sleep, can be quantified with a theoretically motivated measure of the match between neuronal circuits and the environment (*Matching*) (6–8). The benefits are demonstrated here using a integrate-and-fire neuron with six dendritic compartments composed of one feedforward and one feedback connection, with respect to its ability to capture and model the statistical structure of its environment thanks to changes in connection strength. In the companion paper (1), the benefits of potentiation in wake and down-selection in sleep were demonstrated in a parallel set of simulations using neuronal networks engaged in memory consolidation and integration. The results of both approaches indicate that alternative strategies, such as additional synaptic potentiation in wake, potentiation in sleep, or renormalization in wake, lead instead to a decrease in *Matching* (this contribution) and to a worsening of memory performance (1).

Below, we briefly review the theoretical measures employed here and discuss why synaptic homeostasis during sleep turns out to be beneficial for increasing *Matching* and preserving the selectivity of neuronal responses. We also argue that the alternation of synaptic potentiation in wake and depression in sleep, especially when occurring at the level of synaptic clusters, can ensure that different subsets of neurons specialize for different perception-action loops. Moreover, we argue that multiple cycles of potentiation in wake and down-selection in sleep ensure that both long-term features of the environment and new contingencies are integrated within neural circuits, while spurious coincidences, whether external or internal to the system, are systematically eliminated. Thus, the two-step strategy evaluated here represents a sensible way for the brain to address the plasticity-stability dilemma (18, 19).

### 4.1 Matching, selectivity, and S/N

As discussed in Tononi (7, 8) and briefly in the Materials and Methods, measures of *Matching* between a system and its environment can be used to assess how well the system’s connectivity resonates with the causal structure of the environment. *Matching* reflects the ability of the system to capture suspicious coincidences from its inputs and distribute them to many subsets of its units. Moreover, in an integrated system such as the brain, the ability is closely tied to the ability to model the structure of the environment internally (7). Thus, high *Matching* for the brain means that, compared to exposure to noise (a TV screen out of tune), exposure to its environment (a naturalistic movie) will trigger the activation of many different patterns of activity involving many brain areas, which will specify many different constellations of concepts. Moreover, high *Matching* means that, even when the brain is disconnected from the environment (sleep), spontaneous activity will be able to generate experiences (dreams) that will resemble those of wake. In general, *Matching* can be increased both by changing the connections within the system to better match the environment, and by exploring or modifying the environment itself to match the expectations of the system (Albantakis et al., in preparation). For simplicity, the present examples have focused on changes occurring within the system. In the course of evolution, development, and learning, one would expect that the mechanisms of a system change in such a way as to increase *Matching*. This is because, everything else being equal, an organism is better off if it is highly sensitive to the structure of the environment. Moreover, an internal generative model that matches well the overall causal structure of the environment frees the organism from the tyranny of the “here and now,” allowing it to plan ahead and try out imaginary scenarios. Finally, as shown here, the ability to spontaneously activate this internal model during sleep allows the brain to consolidate and integrate memories in a way that, rather than being at the mercy of a day’s limited experiences, takes into account its overall knowledge of the environment.

Ideally, calculating *Matching* exhaustively requires complete knowledge of the architecture of a system, as well as systematic sampling of its possible inputs. Since this is not generally feasible, various heuristics can be employed (Albantakis et al., in preparation; Boly et al., in preparation). On the other hand, in the present case of a single binary neuron, *Matching* can be measured precisely simply by comparing the distribution of firing and silence in the *World* and *Noise* (*Chance*) conditions. In this way, we could examine how learning in wake and down-selection in sleep affect the overall match between a simple neural system and its environment, complementing the result obtained in the companion paper (1) with a focus on specific aspects of memory and performance.

In the above examples, a single neuron learns to sample suspicious coincidences from the environment during wake (for example, the frequent co-occurrence of inputs A and B) by strengthening the corresponding feedforward connections as well as the corresponding feedback connections. Eventually, this produces an increase in *Matching* as compared to a neuron with uniform synaptic strength (Figures 3 and 4). Since the uniform neuron has not tuned its connections to emphasize inputs on A and B, its firing does not show a differential sensitivity to AB inputs, but merely reflects their frequency of occurrence. By contrast, the specialized neuron, having strengthened connections AB, is especially sensitive to AB inputs, and fires for them in proportion to their degree of “suspiciousness.” In richer environments and more complex networks, including those simulated in the companion paper, there will be many more suspicious coincidences, including many of high order (14). Moreover, changes in connection strength during learning in wake will occur at multiple levels and in diverse neural circuits. Nevertheless, as long as such changes produce a differential sensitivity to representative inputs from *World* as compared to *Chance*, *Matching* will increase as shown here.

As also shown here (Figure 5), however, given some reasonable assumptions about limitations in firing rates and synaptic strength, *Matching* will only increase after new learning if synaptic potentiation in wake is followed by synaptic down-selection in sleep. This is because, if learning is not followed by renormalization, while the model neuron will fire more for newly detected suspicious coincidences (e.g., BC, the new “signal”), its average firing rate will increase even more for *Chance* inputs (the “noise”), leading to an overall reduction of *Matching*. This decrease in *Matching* reflects, in the most general terms, the decrease in the S/N of performance indices observed after learning in the companion paper (1), in previous modeling work (20), and in experimental studies of procedural learning (21). On the other hand, activity-dependent down-selection during the sleep phase leads to a marked increase in *Matching*, reflecting an increased differential in the neurons response to suspicious coincidences (signal) vs. spurious ones (noise).

Within the limitations of the present single-neuron model, the above simulations also indicate that alternative strategies for plasticity are not beneficial for *Matching*. As shown in Figure 6, simply adding extra learning in wake does not improve *Matching*, but rather further decreases it. Once again, this result is consistent with a further reduction of S/N observed in simulations (1, 20, 22) and experimentally (21).

Similarly, spontaneous activity accompanied by synaptic potentiation in sleep, even if followed by an overall scaling down of synaptic weights, decreases *Matching* rather than increasing it. This happens because spontaneous activity in the *Sleep* mode is partly according to chance, not being constrained by environmental inputs. If these additional spurious coincidences lead to synaptic potentiation in absolute terms, rather than simply achieving better protection against depression, *Matching* is inevitably reduced. Analogous results were obtained in Nere et al. (1) and in Olcese et al. (22) in terms of memory performance.

The above simulations also indicate that, if synaptic renormalization were to occur in wake, it would disrupt memory traces that should not be obliterated, again leading to a reduction in *Matching*. As argued elsewhere (2–4) and demonstrated in the companion paper (1), this is because a particular episode of wake does not afford a comprehensive sampling of the overall statistical structure of the environment, but forces the organism to learn what is available “here and now.” Under these circumstances, renormalizing synaptic strength would lead to inappropriate forgetting of important memories merely because they do not happen to be engaged by current interactions with the environment. For this reason, it is essential that synaptic renormalization takes place off-line, i.e., during sleep (2–4).

Finally, the present simulations suggest that, if after initial acquisition a particular memory trace is never activated during wake, and if it does not fit well with the rest of the memories incorporated inside the network, it will be erased progressively over many wake-sleep cycles. Such a mechanism allows the brain to slowly forget old memories that do not correspond anymore to relevant features of the environment, thereby increasing *Matching*, in line with neuropsychological results (23). By contrast, activity-dependent down-selection during sleep preferentially protects those new memories that fit with old ones, and are thus frequently co-activated in the *Sleep* mode, at the expense of those that do not. This finding is consistent with both experimental evidence on the role of sleep in memory integration (24, 25) and with the benefits for memory performance observed in the companion paper (1).

### 4.2 Dendritic compartments, specificity of cliques, and perception-action loops

In the present paper, we have considered a single integrate-and-fire neuron and showed that, by employing a two-step strategy of synaptic potentiation in wake and depression in sleep, the neuron could increase its ability to capture suspicious coincidences in its input, as conveyed by feedforward connections and modeled internally through feedback connections. As shown in the companion paper (1), this strategy works not only at the level of the individual neuron, but also at the systems level, leading to memory consolidation, gist extraction, and integration of new with old memories. At the systems level, the benefits of this two-step procedure become even clearer when combined with the provision that plastic changes are sensitive to coincidences within individual dendritic domains (Figure 2). This is because, by taking advantage of distinct dendritic domains, a neuron with a single output (axon) can learn to organize its inputs so as to fire at different times in cooperation with different subsets of other neurons (cliques), engaging in different *perception-action loops* that produce different functions (for other proposals potential advantages of dendritic computation, see for example (26–28)).

**Figure 9:**
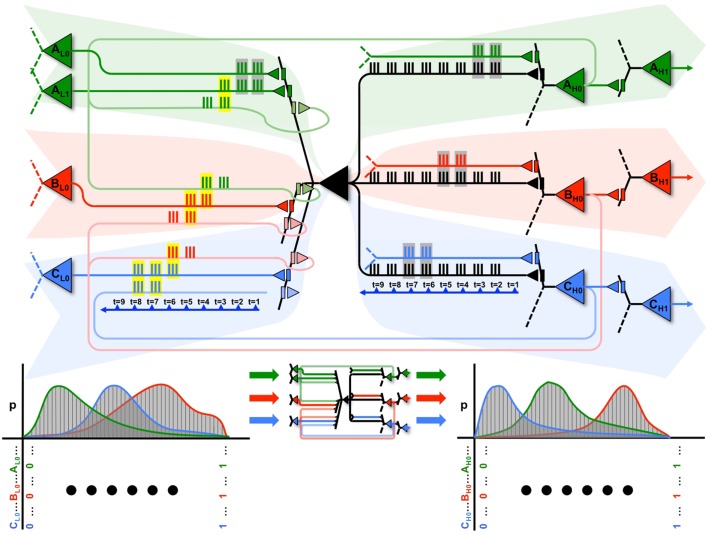
**A neuron can participate in multiple perception-action loops**. Top: through its multiple dendritic domains, a single neuron can participate in multiple perception-action loops. As shown in the figure, the black neuron is part of three different cliques, shown in green, red, and blue. Coincident spiking events are surrounded by gray boxes, while coincident spiking events that cause plastic changes (i.e., both feedforward and feedback spikes) are surrounded by yellow boxes. Bottom: the black neuron may have different cause repertoires when participating with different cliques, as shown by the different red, green, and blue cause repertoires.

This notion is illustrated graphically in Figure 9, where the black neuron (*X*) is part of 3 cliques (A in green, B in red, and C in blue), each associated with a different dendritic compartment. By strengthening subsets of connections from clique A (in green), when a persistent feedforward input that made *X* spike (mediated primarily by AMPA receptors) is associated with persistent feedback activations (mediated primarily by NMDA receptors) on the same dendritic domain, *X* can eventually ensure that firing together with clique A will produce input A, while firing together with a different clique B will produce input B, and so on.

This is illustrated at the top of Figure 9. The propagation of spikes is shown relative to a timeline (blue arrow). Initially, feedforward activations from the green clique (on the left) cause *X* to fire. This, in turn, produces an effect on a downstream green neuron (on the right). Whether directly, or through multiple levels of neurons, this feedback ultimately reaches back to neuron *X*. In the Figure, coincident input spikes in a dendritic domain are boxed in gray, and if coincident inputs contain both feedforward and feedback activation (and thus, induce plasticity) they are boxed in yellow. These closed loops are shown within the red and blue cliques as well. The bottom of Figure 9 shows that, for each different clique the neuron *X* interacts with, it may have a distinct cause repertoire, and in turn, a related effect repertoire on downstream neurons within the same clique.

Furthermore, these perception-action loops can also exist between different cliques. As shown in the figure, the perception-action loop is first closed between the neurons in the green clique, which then prime the perception-action loop of the red clique, which finally prime the perception-action loop of the blue clique. Thus, neuron *X* can participate in sequences of perception-action loops as its input changes.

By following such a local synaptic rule, the neuron will be able to produce different effects for different outputs, thereby ensuring that different cliques of neurons carry out different functions (functional specialization). A local mechanism capable of ensuring functional specialization not just for individual neurons, but for different combinations of neurons (cliques), should provide an economic means to generate a large number of concepts with comparatively few units (7), as required by environments with a rich causal structure. Such an exuberance of available perception-action loops will be reflected in high values of *Matching*, indicating an increased ability to capture correlations in the environment as well as an increased ability to model such correlations internally.

### 4.3 Wake-sleep cycles and the plasticity-stability dilemma

**Figure 10:**
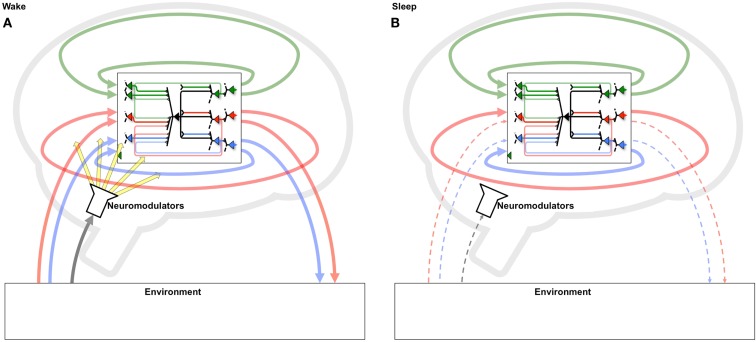
**Systematic potentiation of neural loops including the environment in wake and depression of those excluding it in sleep**. **(A)** During wake, neurons participate in a “grand loop” which includes sensory input from and motor outputs to the outside world. During wake, synaptic potentiation leads to the formation of new memories by strengthening several perception-action loops. **(B)** During sleep, the grand loop with the outside world is interrupted, and only loops internal to the brain can be active. Thus, a systematic process of synaptic potentiation in wake, followed by activity-dependent down-selection in sleep, will enhance circuits that capture important environmental contingencies, especially those that fit with previously formed memories, while progressively eliminating those that do not.

The benefits of a two-step strategy of selective potentiation in wake, guided by environmental constraints and novelty, and down-selection in sleep, guided by internal constraints and accumulated memories, become especially relevant if one considers the regular alternation between states of wake and sleep. During a wake episode, the “grand loop” between the behaving brain and the environment is functioning (Figure 10A). In this way, the multiple brain circuits that are actively involved (selected for) in perception-action loops with that particular environment tend to be potentiated, leading to the incorporation of new memories. During the following sleep episode, the grand loop with the environment is interrupted, the brain goes off-line, and the loops internal to the brain are activated spontaneously, disconnected from the contingencies of a particular environment (Figure 10B). Instead, internal loops are activated in a comprehensive manner, calling into play a vast body of old memories. Moreover, plasticity switches to a mode where internal loops that are most activated are protected rather than potentiated, while other loops are depressed (selected against). As suggested by the present and the companion paper, repeated cycles of positive selection favoring novelty in wake, coupled with negative selection protecting consistent memories in sleep, should lead to two related benefits: (i) the progressive incorporation of environmental contingencies that are consistently present in the environment and/or fit the overall structure of knowledge, and (ii) the progressive elimination of those that are spurious, do not fit previous knowledge, or represent “fantasies” generated by free-running neural circuits. Over multiple cycles, such a dual process offers a potential solution to the classic plasticity-stability dilemma: a learning system should be plastic enough to incorporate new information, but stable enough to preserve important memories over time: that is, learning new associations should not wipe out previous learned ones (18, 19). This is especially true of a system, such as the brain, in which the majority of elements connect to other elements within the system rather than directly to the environment. Such an organization has the advantage of allowing the brain’s actions to be guided by memory (intrinsic models) and thereby go far beyond the current sensory evidence (6, 29). On the other hand, in such systems it becomes important to ensure that the memories continue to match the environment. As shown here, this can be done by systematically selecting in favor of the concepts generated when the system is embedded in the grand loop that includes the external environment (wake), and against those produced by the system in isolation (sleep/dreaming).

## 5 Conclusion

The synaptic homeostasis hypothesis (2–4), investigated here as well as in a companion paper (1), postulates that memory functions utilize a two-step process: in wake, neurons learn to capture suspicious coincidences in the environment predominately through synaptic potentiation; in sleep, when disconnected from the outside world, a neuron samples the memories it has formed and synapses it has strengthened and uses activity-dependent down-selection to selectively depress circuits. Unlike other models which allow further synaptic potentiation during memory reactivation in sleep, the two-step process proposed by the synaptic homeostasis hypothesis demonstrates all the associated benefits of sleep on memory, without the risk of forming “spurious” or “fantasy” memories in sleep. In this paper, we investigated the benefits of synaptic down-selection during sleep using a single, simplified neuron model and the theoretical measure of *Matching* which assesses how well a neuron is tuned to a particular environment. Furthermore, we demonstrated how extended wake, potentiation in sleep, and down-selection in wake each can reduce the measure of *Matching*, providing complementary evidence to the systems level simulations presented in a companion paper (1).

## Conflict of Interest Statement

The authors declare that the research was conducted in the absence of any commercial or financial relationships that could be construed as a potential conflict of interest.

## References

[1] Nere AT Hashmi A Cirelli C Tononi G Sleep dependent synaptic down-selection (I): modeling the benefits of sleep on memory consolidation and integration. Front Neurol (2013) 4:143 10.3389/fneur.2013.00143 24137153 PMC3786405

[2] Tononi G Cirelli C Sleep and synaptic homeostasis: a hypothesis. Brain Res Bull (2003) 620(2):143–50 10.1016/j.brainresbull.2003.09.004 14638388

[3] Tononi G Cirelli C Sleep function and synaptic homeostasis. Sleep Med Rev (2006) 100(1):49–62 10.1016/j.smrv.2005.05.002 16376591

[4] Tononi G Cirelli C Time to be SHY? Some comments on sleep and synaptic homeostasis. Neural Plast (2012) 2012:415250 10.1155/2012/415250 22619736 PMC3350977

[5] Balduzzi D Tononi G What can neurons do for their brain? Communicate selectivity with bursts. Theory Biosci (2013) 1320(1):27–39 10.1007/s12064-012-0165-0 22956291 PMC3549471

[6] Tononi G Sporns O Edelman GM A complexity measure for selective matching of signals by the brain. Proc Natl Acad Sci U S A (1996) 930(8):3422–7 10.1073/pnas.93.8.3422 8622951 PMC39624

[7] Tononi G Integrated information theory of consciousness: an updated account. Arch Ital Biol (2012) 1500(2-3):56–90 23165867 10.4449/aib.v149i5.1388

[8] Tononi G On the irreducibility of consciousness and its relevance to free will. In: Suarez A Adams P editors. Is Science Compatible with Free Will? New York: Springer (2013). p. 147–76

[9] Attwell D Laughlin SB An energy budget for signaling in the grey matter of the brain. J Cereb Blood Flow Metab (2001) 210(10):1133–45 10.1097/00004647-200110000-00001 11598490

[10] Vyazovskiy VV Cirelli C Pfister-Genskow M Faraguna U Tononi G Molecular and electrophysiological evidence for net synaptic potentiation in wake and depression in sleep. Nat Neurosci (2008) 110(2):200–8 10.1038/nn2035 18204445

[11] Self MW Kooijmans RN Supèr H Lamme VA Roelfsema PR Different glutamate receptors convey feedforward and recurrent processing in macaque v1. Proc Natl Acad Sci U S A (2012) 1090(27):11031–6 10.1073/pnas.1119527109 22615394 PMC3390882

[12] Jones BE Chapter 11 - basic mechanisms of sleep-wake states. 4th ed In: Kryger MH Roth T Dement WC editors. Principles and Practice of Sleep Medicine. Philadelphia: W.B. Saunders (2005). p. 136–53

[13] Watson CJ Baghdoyan HA Lydic R Neuropharmacology of sleep and wakefulness. Sleep Med Clin (2010) 50(4):0513 10.1016/j.jsmc.2010.08.003 PMC3026477 21278831

[14] Barlow HB The twelfth Bartlett memorial lecture: the role of single neurons in the psychology of perception. Q J Exp Psychol (1985) 370(2):121–45 10.1080/14640748508400927 2991994

[15] Kudrimoti HS Barnes CA McNaughton BL Reactivation of hippocampal cell assemblies: effects of behavioral state, experience, and EEG dynamics. J Neurosci (1999) 190(10):4090–101 10234037 10.1523/JNEUROSCI.19-10-04090.1999 PMC6782694

[16] Nádasdy Z Hirase H Czurkó A Csicsvari J Buzsáki G Replay and time compression of recurring spike sequences in the hippocampus. J Neurosci (1999) 190(21):9497–507 10531452 10.1523/JNEUROSCI.19-21-09497.1999 PMC6782894

[17] Ji D Wilson MA Coordinated memory replay in the visual cortex and hippocampus during sleep. Nat Neurosci (2007) 100(1):100–7 10.1038/nn1825 17173043

[18] Grossberg S Competitive learning: from interactive activation to adaptive resonance. Cogn Sci (1987) 110(1):23–63 10.1111/j.1551-6708.1987.tb00862.x

[19] Abraham WC Robins A Memory retention – the synaptic stability versus plasticity dilemma. Trends Neurosci (2005) 280(2):73–8 10.1016/j.tins.2004.12.003 15667929

[20] Hill S Tononi G Ghilardi MF Sleep improves the variability of motor performance. Brain Res Bull (2008) 760(6):605–11 10.1016/j.brainresbull.2008.02.024 18598851 PMC2494731

[21] Huber R Ghilardi MF Massimini M Tononi G Local sleep and learning. Nature (2004) 4300(6995):78–81 10.1038/nature02663 15184907

[22] Olcese U Esser SK Tononi G Sleep and synaptic renormalization: a computational study. J Neurophysiol (2010) 1040(6):3476–93 10.1152/jn.00593.2010 20926617 PMC3007640

[23] Hardt O Nader K Nadel L Decay happens: the role of active forgetting in memory. Trends Cogn Sci (2013) 170(3):111–20 10.1016/j.tics.2013.01.001 23369831

[24] Diekelmann S Born J The memory function of sleep. Nat Rev Neurosci (2010) 110(2):114–26 20046194 10.1038/nrn2762

[25] Stickgold R Walker MP Sleep-dependent memory triage: evolving generalization through selective processing. Nat Neurosci (2013) 160(2):139–45 10.1038/nn.3303 23354387 PMC5826623

[26] Polsky A Mel BW Schiller J Computational subunits in thin dendrites of pyramidal cells. Nat Neurosci (2004) 70(6):621–7 10.1038/nn1253 15156147

[27] Behabadi BF Polsky A Jadi M Schiller J Mel BW Location-dependent excitatory synaptic interactions in pyramidal neuron dendrites. PLoS Comput Biol (2012) 8(7):e1002599 10.1371/journal.pcbi.1002599 22829759 PMC3400572

[28] Legenstein R Maass W Branch-specific plasticity enables self-organization of nonlinear computation in single neurons. J Neurosci (2011) 310(30):10787–802 10.1523/JNEUROSCI.5684-10.2011 21795531 PMC6623094

[29] Tononi G Edelman GM Information: in the stimulus or in the context? Behav Brain Sci (1997) 20:698–700 10.1017/S0140525X97401607

